# Acidic Chitinase-Chitin Complex Is Dissociated in a Competitive Manner by Acetic Acid: Purification of Natural Enzyme for Supplementation Purposes

**DOI:** 10.3390/ijms19020362

**Published:** 2018-01-25

**Authors:** Eri Tabata, Akinori Kashimura, Satoshi Wakita, Masayoshi Sakaguchi, Yasusato Sugahara, Yasutada Imamura, Hideaki Shimizu, Vaclav Matoska, Peter O. Bauer, Fumitaka Oyama

**Affiliations:** 1 Department of Chemistry and Life Science, Kogakuin University, Hachioji, Tokyo 192-0015, Japan; bm16024@ns.kogakuin.ac.jp (E.T.); bu41265@ns.kogakuin.ac.jp (A.K.); bd15003@ns.kogakuin.ac.jp (S.W.); bt11532@ns.kogakuin.ac.jp (M.S.); bt79310@ns.kogakuin.ac.jp (Y.S.); bt40522@ns.kogakuin.ac.jp (Y.I.); 2 RIKEN Center for Life Science Technologies, Tsurumi, Yokohama 230-0045, Japan; hideaki.shimizu@riken.jp; 3 Department of Clinical Biochemistry, Hematology and Immunology, Homolka Hospital, 142 20 Prague, Czech Republic; vaclav.matoska@homolka.cz (V.M.); peter.bauer@bioinova.cz (P.O.B.); 4 Bioinova Ltd., 142 20 Prague, Czech Republic

**Keywords:** acetic acid, acidic chitinase, chitin, chitin column, competitive manner, GlcNAc, natural enzyme, supplementation purposes, therapeutic agents, urea

## Abstract

Acidic chitinase (Chia) has been implicated in asthma, allergic inflammations, and food processing. We have purified Chia enzymes with striking acid stability and protease resistance from chicken and pig stomach tissues using a chitin column and 8 M urea (urea-Chia). Here, we report that acetic acid is a suitable agent for native Chia purification from the stomach tissues using a chitin column (acetic acid-Chia). Chia protein can be eluted from a chitin column using 0.1 M acetic acid (pH 2.8), but not by using Gly-HCl (pH 2.5) or sodium acetate (pH 4.0 or 5.5). The melting temperatures of Chia are not affected substantially in the elution buffers, as assessed by differential scanning fluorimetry. Interestingly, acetic acid appears to be more effective for Chia-chitin dissociation than do other organic acids with similar structures. We propose a novel concept of this dissociation based on competitive interaction between chitin and acetic acid rather than on acid denaturation. Acetic acid-Chia also showed similar chitinolytic activity to urea-Chia, indicating that Chia is extremely stable against acid, proteases, and denaturing agents. Both acetic acid- and urea-Chia seem to have good potential for supplementation or compensatory purposes in agriculture or even biomedicine.

## 1. Introduction

Chitin is a polymer of (β-1,4)-linked *N*-acetyl-d-glucosamine (GlcNAc), which is an integral component of the exoskeleton of crustaceans and insects, microfilarial sheaths of parasites, and cell walls in fungi [1,2,3]. Chitin is insoluble in water, mild acidic or basic solutions, and organic solvents due to its intermolecular hydrogen bonds [1,3,4,5].

Chitinases are important enzymes responsible for chitin metabolism in a wide range of organisms, including bacteria, fungi, nematodes, and arthropods [2,6,7]. Mammals, including mice and humans, do not synthesize chitin; however, they do express two active chitinases: chitotriosidase (Chit1) and acidic chitinase (Chia) (hereafter referred to as “Chia”; alternative name: acidic mammalian chitinase, AMCase) [7,8].

Chia is highly expressed in mouse stomach tissues and is most active at pH 2.0 [9,10,11,12]. Chia is a digestive enzyme that breaks down chitin while being a part of the host against chitin-containing pathogen defense in the stomach [9,10,11,12,13,14]. Recently, we have shown that Chia enzymes function as major protease-resistant glycosidases under gastrointestinal tract (GIT) conditions in mice, chickens, and pigs [15,16,17].

Significant increases in Chia mRNA and protein levels have been detected in an induced asthma as well as in an antigen-induced allergic lung inflammation in mouse models [18,19]. In addition, it has been shown that single nucleotide polymorphisms in human Chia are associated with asthma [20,21,22].

In contrast, decreases in Chia mRNA and protein levels accompany dry eye syndrome and gastric cancer [23,24,25,26,27]. In the lung of Chia-deficient mice, environmentally derived chitin polymers are accumulated in the airways, inducing spontaneous pulmonary fibrosis [28]. In addition, the chitinolytic activity of the human Chia is significantly lower than that of the mouse Chia [22]. We have found that not all animals abundantly express Chia. Specifically, herbivorous and carnivorous animals such as bovines and dogs have very low capability to digest chitin when compared with omnivorous animals [29]. This knowledge could potentially lead to the development of Chia-related supplementation for chitin digestion enhancement or as promising therapeutic agents for the treatment of specific diseases resulting from low levels and/or activity of Chia.

For supplementation purposes, large quantities of natural purified enzyme are required. Many chitinases have been reported to be isolated from bacteria, plants, and animals [30,31,32,33,34,35,36,37,38]. We recently reported that Chia can be purified from chicken and pig stomach tissues using a chitin column and 8 M urea [16,17]. Several chitinases have been eluted from a chitin column using acetic acid but the mechanism of the elution has not been investigated [31,32,33,34,35,36,38]. Acetic acid is a weak acid, but can cause acid denaturation of common chitinases. Chicken and pig Chia proteins have remarkable acid stability [16,17].

Here, we report that a Chia-chitin complex is dissociated in a competitive manner by 0.1 M acetic acid. This acetic acid competition toward chitin can be applied to the purification of endogenous chicken and pig Chia, potentially having numerous applications for agricultural or biomedical Chia-related supplementation purposes.

## 2. Results

### 2.1. Dissociation of Chicken Chia–Chitin Complex by 0.1 M Acetic Acid

**Figure 1:**
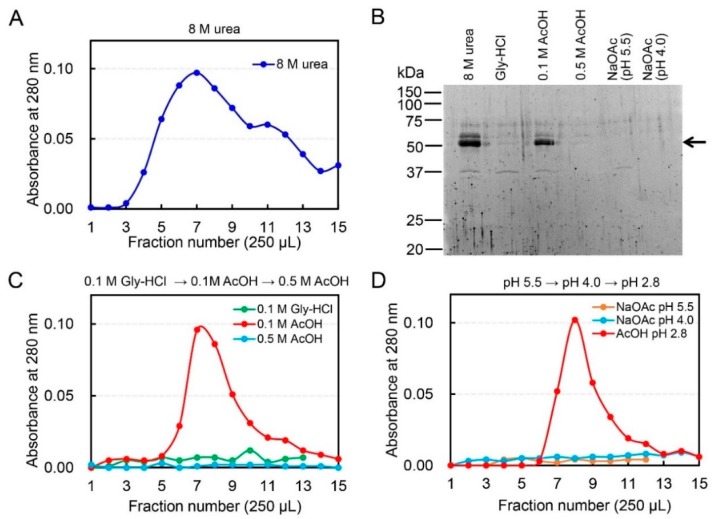
Specific dissociation of chicken acidic chitinase (Chia)-chitin by 0.1 M acetic acid. (**A**) Elution profiles of chicken Chia from chitin column by 8 M urea. The soluble fraction of chicken glandular stomach was applied onto the column. Bound chitinase was eluted from the column with 8 M urea. (**B**) SDS-PAGE analysis of the protein fractions. Arrow highlights the positions of the Chia protein. (**C**) Elution profiles of Chia from chitin column by 0.1 M Gly-HCl (pH 2.5) or 0.1 M acetic acid (pH 2.8). The column was further washed with 0.5 M acetic acid. (**D**) Elution of bound Chia by 0.1 M sodium acetate (pH 4.0 or pH 5.5). Bound Chia was finally eluted from chitin column by 0.1 M acetic acid.

We purified Chia by application of chicken stomach extract onto the chitin beads column using several elution conditions. The enzyme was eluted as a single peak with 8 M urea. The eluted fractions were analyzed by SDS-polyacrylamide gel electrophoresis (PAGE) and SYPRO Ruby staining. SDS-PAGE analysis showed one major and one minor band at 54 and 57 kDa, respectively (Figure 1B), consistent with our recent report [16].

Next, we examined whether the Chia-chitin complex can be dissociated by 0.1 M Gly-HCl (pH 2.5) or 0.1 M acetic acid (pH 2.8). Chia was eluted by the acetic acid but not by Gly-HCl (Figure 1B,C). The 0.1 M acetic acid-treated column was further exposed to 0.5 M acetic acid. There was no further protein elution detected (Figure 1B,C) indicating that 0.1 M acetic acid is sufficient for the complete detachment of the Chia enzyme from the chitin column. These results suggested that the elution of Chia protein from the chitin column is not caused by acid denaturation, but rather by the competitive binding of acetic acid.

We speculated that the elution of Chia from chitin could result from a competition between GlcNAc and acetate ions. To test this possibility, we carried out elution by 0.1 M sodium acetate at pH 4.0 and pH 5.5. As shown in Figure 1B,D, no protein was eluted by 0.1 M sodium acetate (pH 4.0 and pH 5.5). In contrast, Chia was eluted from the chitin column with 0.1 M acetic acid at pH 2.8. These results suggest that Chia is not eluted by competition of GlcNAc with acetate ions, but by acetic acid.

### 2.2. Acetic Acid Is Suitable for Chicken Chia Elution from Chitin Column without Acid Denaturation

**Figure 2:**
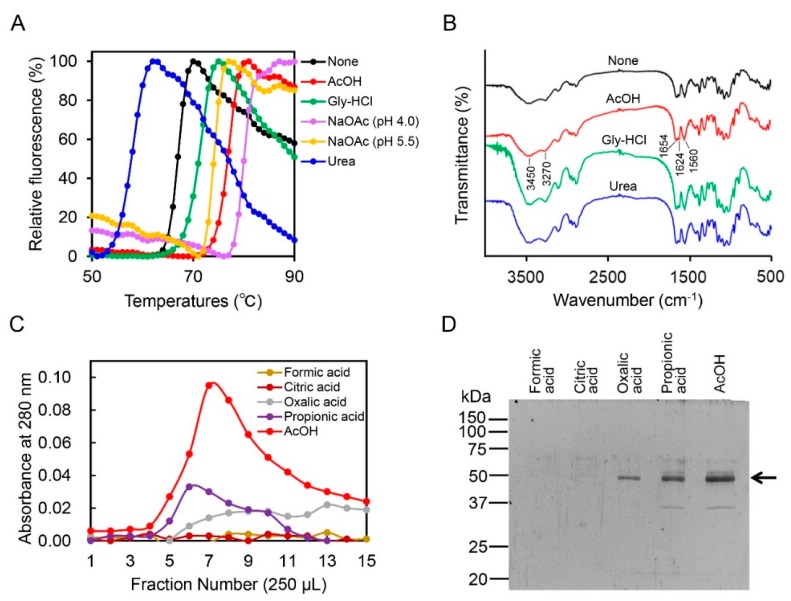
Evaluation of Chia-chitin dissociation by effects of Gly-HCl buffer, acetic acid, sodium acetate, and urea and their effect on the enzyme and chitin structure. (**A**) DSF analysis results for Chia in the presence of 0.1 M Gly-HCl, 0.1 M acetic acid, 0.1 M sodium acetate (pH 4.0 and pH 5.5), and 4 M urea are shown. (**B**) FT-IR spectra of chitin after treatment with water (none), acetic acid (AcOH), Gly-HCl, and urea. (**C**) Elution profiles of Chia from chitin column by 0.1 M formic acid (pH 2.5), 0.1 M citric acid (pH 2.5), 0.1 M oxalic acid (pH 2.5), 0.1 M propionic acid (pH 2.5), or 0.1 M acetic acid (pH 2.8). (**D**) SDS-PAGE analysis of the protein fractions. Arrow highlights the positions of the Chia protein.

We investigated the mechanism of Chia-chitin complex dissociation by acetic acid in terms of structural alteration of the enzyme and chitin (Figure 2A,B). To monitor the melting temperatures (Tm) of proteins corresponding to solution-dependent changes in protein stability, differential scanning fluorimetry (DSF) [39] was performed. The values for the Tm were 70.9, 76.7, 80.0, and 74.1 °C in the presence of 0.1 M Gly-HCl (pH 2.5), 0.1 M acetic acid (pH 2.8), and 0.1 M sodium acetate (pH 4.0 and pH 5.5), respectively. In contrast, 4 M urea reduced the stability of the protein markedly and changed the values to 56.5 °C (Figure 2A). These results indicated that Chia was not denatured by acetic acid, Gly-HCl, or sodium acetate.

Next, we analyzed the alteration of the buffer-treated chitin by Fourier transform infrared (FT-IR) spectroscopy as described in Materials and Methods. The spectra bands of α-chitin treated with Gly-HCl, acetic acid, or urea were similar to those of chitin treated with water (Figure 2B). The four infrared spectra showed the characteristic bands of the chitin molecule: O-H stretching (3450 cm^−1^), N-H stretching (3270 cm^−1^), amide I (1654 and 1624 cm^−1^), and amide II (1560 cm^−1^) [40]. Thus, these spectra indicate that acetic acid as well as Gly-HCl and urea do not change the chitin structure.

To further investigate the dissociation mechanism of the Chia-chitin complex by acetic acid, Chia was eluted from the chitin column by several organic acids including formic acid, citric acid, oxalic acid, and propionic acid, which each have a similar functional group to that of acetic acid. While formic acid and citric acid could not elute the protein, it was weakly eluted by oxalic acid and propionic acid (Figure 2C,D). Acetic acid, on the other hand, eluted the enzyme with a superior efficiency as compared to other organic acids (Figure 2C,D).

### 2.3. Effect of Acetic Acid on Chitin Degradation by Chicken Chia Enzyme

**Figure 3:**
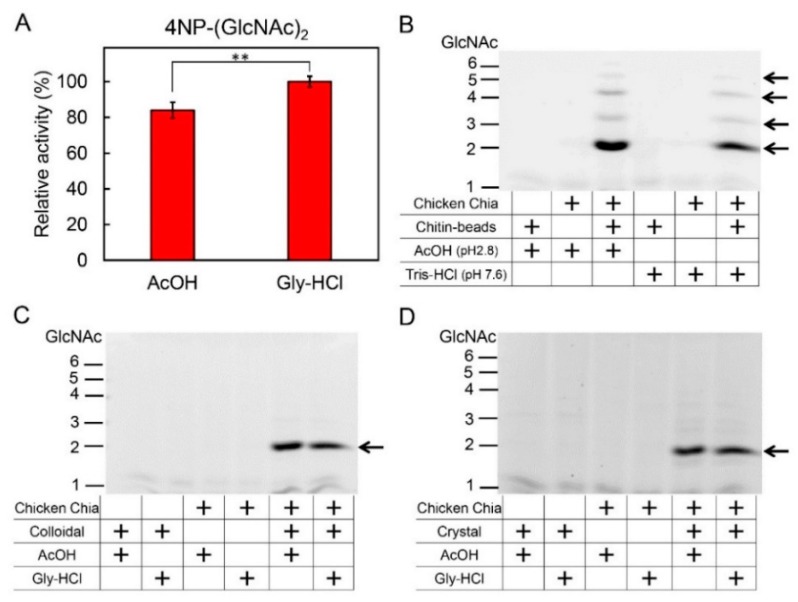
Effect of acetic acid on chitin degradation. (**A**) The chitinolytic activity of Chia was analyzed using 4-NP-(GlcNAc)_2_ at pH 2.8 using 0.1 M Gly-HCl or 0.1 M acetic acid. The absorbance of the liberated 4-nitrophenolate ion was measured at 405 nm. ** p  <  0.01. *p*-Values were determined using Student’s *t*-test. (**B**) Degradation of chitin bound to beads by purified chicken Chia under 0.1 M acetic acid (pH 2.8) or 0.1 M Tris-HCl (pH 7.6) conditions. Produced oligosaccharides were analyzed by the fluorophore-assisted carbohydrate electrophoresis (FACE) method. Arrows indicate the positions of (GlcNAc)_2_, (GlcNAc)_3_, (GlcNAc)_4_, and (GlcNAc)_5_. Degradation products generated by incubation of (**C**) colloidal or (**D**) crystalline chitin with chicken Chia at pH 2.8 using 0.1 M Gly-HCl or 0.1 M acetic acid. Degradation products were analyzed by the FACE method. Arrow indicates the positions of (GlcNAc)_2_.

Next, we investigated the competition between acetic acid and chitin substrates in terms of binding to Chia. We assessed the effects of this competition on chitin degradation by Chia using artificial and natural chitin substrates. We first incubated 4-nitrophenyl *N*,*N′*-diacetyl-β-d-chitobioside (4-NP-chitobioside) with chicken Chia at pH 2.8 using 0.1 M Gly-HCl or 0.1 M acetic acid. The chitinolytic activity in 0.1 M acetic acid was slightly lower than that in 0.1 M Gly-HCl (pH 2.8) (Figure 3A).

Similarly, we analyzed this effect on chitin degradation. We incubated chitin beads with or without purified Chia and 0.1 M acetic acid at pH 2.8 or 0.1 M Tris-HCl at pH 7.6. Mono- and oligosaccharides produced from chitin beads in the reaction were analyzed by fluorophore-assisted carbohydrate electrophoresis (FACE) [41,42]. Although chitin on the beads was degraded primarily to (GlcNAc)_2_ with several GlcNAc oligomers by chicken Chia, none of the specific bands were detected in the reaction mixture by incubation without Chia (Figure 3B). These results indicate that chitin was not degraded by 0.1 M acetic acid (pH 2.8) or 0.1 M Tris-HCl (pH 7.6), but could be degraded by Chia under both pH conditions (Figure 3B). These data also suggest that the elution of Chia from the chitin column does not occur due to acid denaturation, but due to the competition by acetic acid.

To further analyze the effect of acetic acid on the degradation of the natural chitin substrates, we incubated colloidal and crystalline chitin with chicken Chia in 0.1 M Gly-HCl (pH 2.8) or 0.1 M acetic acid (pH 2.8). In contrast to artificial substrates, there was no significant difference between the buffer conditions. Chia degraded both chitin substrates and generated primarily (GlcNAc)_2_ fragments at a similar rate (Figure 3C,D). These results suggest that acetic acid dissociates the Chia-chitin complex but does not inhibit its hydrolytic activity.

### 2.4. CatD and CBD of Chicken Chia Bind with Chitin Column, Which Were Eluted by Acetic Acid

**Figure 4:**
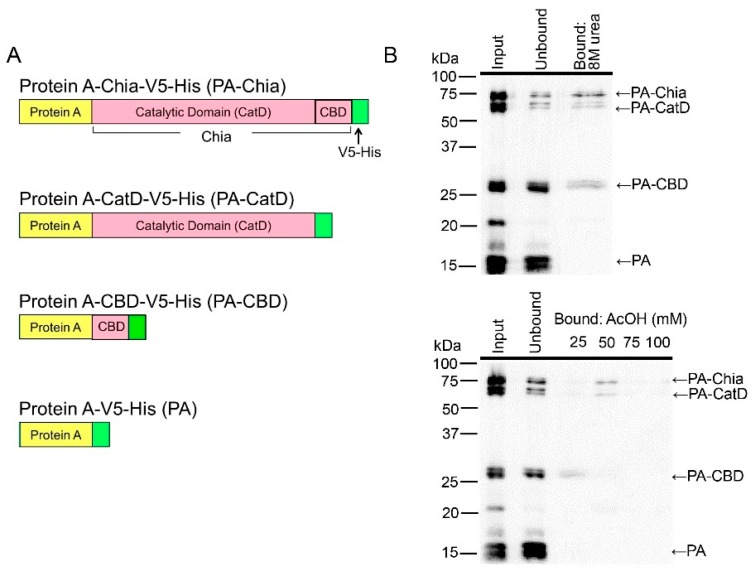
Binding of recombinant chicken full-length Chia as well as its catalytic domain (CatD) and chitin binding domain (CBD) are dissociated by acetic acid. (**A**) The schematic representations of the recombinant chicken Chia fusion proteins. (**B**) Western blot analysis of the recombinant proteins using anti-V5 antibody. Upper panel; Mixtures of the recombinant proteins were loaded onto a single chitin column. Bound proteins were eluted from the column with 8 M urea. Lower panel; Western blot analysis of the recombinant proteins. Bound proteins were eluted from the column with 25, 50, 75, and 100 mM acetic acid (stepwise elution). Arrows highlight the position of each fusion protein.

To determine the affinity of the catalytic domain (CatD) or chitin binding domain (CBD) of chicken Chia to the chitin column, we expressed full-length Chia, CatD, and CBD in *E. coli* as fusion proteins with a truncated form of *Staphylococcus aureus* Protein A and V5-His tag [12,14,22] as described in Materials and Methods. The schematic representations of these recombinant proteins are shown in Figure 4A (see also Figure S1).

To determine whether the Protein A fusion proteins can recognize and interact with chitin, we carried out a binding assay using a chitin bead column. We mixed Protein A-Chia-V5-His (PA-Chia), Protein A-CatD-V5-His (PA-CatD), Protein A-CBD-V5-His (PA-CBD), and Protein A-V5-His (PA) at pH 7.6 and loaded the samples onto a single column as described in Materials and Methods. Proteins capable of binding to chitin were eluted from the column with 8 M urea (Figure 4B, upper panel). PA-Chia, PA-CatD, and PA-CBD can bind to the chitin column, whereas PA was detected only in the flow-through fraction (Figure 4B, upper panel). These were consistent with mouse Chia [14].

Next, we investigated the differences in chitin affinity level among PA-Chia, PA-CatD, and PA-CBD. As shown in Figure 4B, PA-CBD was eluted by 25 mM acetic acid, whereas PA-Chia and PA-CatD were eluted by 50 mM acetic acid (Figure 4B, lower panel). These results clearly indicate that the CatD of Chia proteins has more affinity to the chitin column than the CBD.

### 2.5. Pig Chia Purification from Stomach Tissues Using a Chitin Column and Acetic Acid

**Figure 5:**
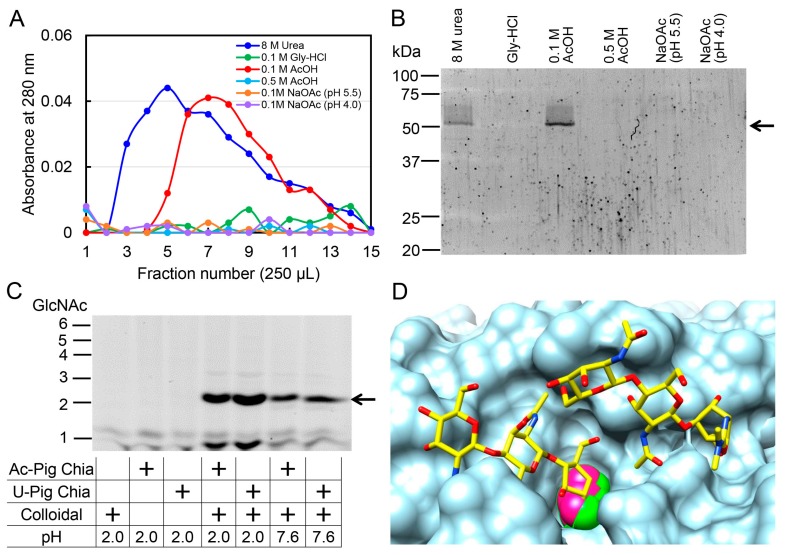
Native Chia can be purified from pig stomach tissues using a chitin column and acetic acid. The soluble fraction of pig stomach was applied onto the column. The bound Chia was eluted from the column as described in Figure 1. (**A**) Elution profiles of pig Chia from the chitin column. (**B**) SDS-PAGE analysis of the protein fractions. Arrow highlights the positions of the Chia protein. (**C**) Comparison of the chitinolytic properties between acetic acid-Chia and 8 M urea-Chia. Colloidal and crystalline chitin substrates were digested by Chia and resulting products were analyzed by the FACE method. Arrow indicates the position of (GlcNAc)_2_. (**D**) Composite surface representation of the chitinase (PDB ID: 3N12) with the superimposed inhibitor allosamidin (PDB ID: 1HKK, yellow-stick model). The acetic acid molecules of 3N12 (pink) and 4NZC (green) are shown at van der Waals radii.

Finally, we applied the above process to prepare the Chia enzyme from pig stomach tissues. Similarly to chicken Chia (Figure 1), pig Chia was specifically eluted from the chitin column by 0.1 M acetic acid with one band at 54 kDa (Figure 5A,B). This was consistent with our recent report using a chitin column and 8 M urea [17].

To compare the chitinolytic properties between the pig Chia-acetic acid and 8 M urea-Chia, polymeric chitin was digested and the resulting products were analyzed by FACE. We detected (GlcNAc)_2_ from the colloidal chitin at pH 2.0 and 7.6, indicating that pig Chia enzymes can function under neutral as well as acidic conditions (Figure 5C).

## 3. Discussion

We have reported efficient purification of Chia enzymes from chicken and pig stomachs using chitin columns and 8 M urea, followed by characterization of their optimal conditions and protease resistances [16,17]. Native Chia proteins have good potential for use as compensatory enzymes for chitin digestion or possible therapeutic agents for certain diseases, such as asthma. Here, we report a suitable method for native Chia purification using a chitin column and acetic acid. Meanwhile, we suggest a novel concept of a Chia-chitin complex being dissociated not by acid denaturation but by competition between chitin and acetic acid. Acetic acid-Chia has potential for application for agricultural and biomedical purposes.

In a previous report, we showed that recombinant Chia protein (Protein A-mouse Chia-V5-His) with chitinolytic activity was eluted from IgG Sepharose by 0.1 M Gly-HCl (pH 2.5) or Ni Sepharose columns by 8 M urea, which causes protein denaturation [12,14,22]. In this study, we applied the same buffers for native Chia preparation using a chitin column. Chia was efficiently eluted by 8 M urea, but not by Gly-HCl (pH 2.5), suggesting that Chia was not denatured by Gly-HCl (pH 2.5) and remained within the chitin column.

Chitinase-chitin interaction was used for the purification of chitinases from a variety of organisms [30,31,32,33,34,35,36,37,38]. Among these interactions, chitinase sometimes irreversibly binds to the chitin substrate and cannot be eluted under nondenaturing conditions. Indeed, Chit1 CBD domain elution from the chitin beads required stringent conditions, such as 1% SDS or 50% acetonitrile [43]. In contrast, some chitinases bind mildly to the chitin column and can be eluted by acetic acid or sodium acetate. A variety of pH values in acetate buffers have been used for chitinase elution from columns [31,32,33,34,35,38]. We first tested 0.1 M Gly-HCl (pH 2.5) or acetic acid (pH 2.8) as well as sodium acetate (pH 4.0 and 5.5) for Chia elution. While Gly-HCl and sodium acetate were not effective, Chia was eluted by 0.1 M acetic acid. Also, oxalic acid and propionic acid, which have similar structures to acetic acid, also eluted Chia from chitin column, but with much lower efficacy. These results imply that interaction between Chia and chitin is specifically affected by carboxyl and/or methyl groups of acetic acid and suggest chitin-acetic acid competition. As far as we know, this is the first report to examine its elution mechanism from the column.

Chia and Chit1 show sequence homology to bacterial chitinases and belong to Family 18 of the glycoside hydrolases (GH18) [6,8,44,45]. Although the overall crystal structure of Chia has remained unresolved, our observations can be applied to the example of related GH18 enzymes. As shown in Figure 5D, the binding site for the substrate has been observed to be occupied by the acetic acid molecule in several crystal structures of the chitinases [46,47,48]. It is therefore likely that the acetic acid molecule has an ability to accommodate at this position, and could consequently interfere with the chitin binding.

Chicken and pig Chia enzymes prepared by 8 M urea (urea-Chia) have acid stability and protease resistance and can degrade chitin into (GlcNAc)_2_ under GIT conditions [16,17]. Even though the Chia proteins were denatured by urea, they still maintained a remarkable enzymatic function. Native Chia enzymes prepared by acetic acid (acetic acid-Chia) also showed similar chitinolytic activity to urea-Chia (Figure 5C). This means that Chia is extremely stable against acid, proteases, and denaturing agents. Thus, acetic acid can be used as an alternative method for purifying natural Chia enzymes using a chitin column.

In food processing industries, chicken egg lysozyme is applied as a natural preservative due to its antibacterial activity [49]. We have reported that chicken and pig Chia enzymes can degrade chitin-containing organisms under their GIT conditions [16,17]. Furthermore, we have shown that Chia mRNA levels were significantly lower in bovine (herbivores) and dog (carnivores) stomach than those in mouse, pig, and chicken (omnivores), indicating that feeding behavior affects Chia mRNA expression levels and determines chitin digestibility in particular animals [29]. Thus, acetic acid- and urea-Chia enzymes are an ideal additive for chitin-containing food to enhance the digestibility of chitin, preventing sickness resulting from chitin overdose in herbivores and carnivores such as bovines and dogs, respectively.

Recently, Chia has become an attractive molecule for potential biomedical purposes. In Chia-deficient mice, environmentally derived chitin polymers accumulated in the airways, resulting in profibrotic cytokine expression induction [28]. These mice develop spontaneous pulmonary fibrosis, which is ameliorated by restoration of lung chitinase activity by different genetic or therapeutic approaches. However, the expression level and chitinolytic activity of human Chia are much lower than those of mouse Chia [13,22]. In addition, significant decreases in Chia mRNA and protein levels have been detected in several pathological conditions [23,24,25,26,27]. Pigs are used as a common animal model in biomedicine due to several similarities with humans in terms of genetics [50], digestive systems, and metabolisms [51]. In addition, pig proteins have been used for compensatory treatments in humans. For instance, porcine insulin has been used for treatment of diabetes [52]. Several diseases which show decreased Chia mRNA and protein levels might be at least partly improved by Chia proteins from pigs or chickens. The purified natural enzymes also do not possess additional sequences like tagged recombinant proteins. Thus, acetic acid- and urea-Chia enzymes may indeed have potential as therapeutic enzymes.

## 4. Materials and Methods

### 4.1. Chicken Glandular Stomach and Pig Stomach Tissues

We purchased normal chicken glandular stomach and pig stomach tissues from Funakoshi Co., Ltd. (Tokyo, Japan) as described previously [16,17].

### 4.2. Preparation of Soluble Proteins from Chicken Glandular Stomach and Pig Stomach

Chicken glandular (1 g) or pig (1 g) stomach tissue was homogenized in 10 volumes of ice-cold TS buffer (20 mM Tris-HCl (pH 7.6), 150 mM NaCl) containing protease inhibitor (Complete Mini, Roche Diagnostics, Mannheim, Germany) using a Teflon/glass homogenizer, followed by centrifugation at 15,000× *g* for 10 min at 4 °C. The supernatants were used in further experiments.

### 4.3. Elution of Chia Proteins from Chitin Column

Chitin columns were prepared by mixing 2 mL of chitin beads (New England Biolabs, Ipswich, MA, USA), followed by equilibration with TS buffer. The extracts were applied, and the columns were sealed and gently inverted at 4 °C for 1 h.

In our preliminary experiments, after extensive washing with TS buffer, the bound protein was eluted with 0.1 M Gly-HCl (pH 2.5), 0.1 M CH_3_COOH (pH 2.8), 0.1 M CH_3_COONa (pH 4.0), 0.1 M CH_3_COONa (pH 5.5), 0.1 M formic acid (pH 2.5), 0.1 M citric acid (pH 2.5), 0.1 M oxalic acid (pH 2.5), or 0.1 M propionic acid (pH 2.5), followed by neutralization with 1 M Tris-HCl (pH 7.6).

### 4.4. Purification of Chicken and Pig Chia Using 8 M Urea or 0.1 M Acetic Acid

The Chia enzymes were purified from chicken glandular stomach or pig stomach tissue using a chitin bead column and eluted with 8 M urea, as described previously [16,17].

The enzymes were also eluted from the columns with 0.1 M acetic acid. The eluted enzymes were neutralized and desalted with PD10 (GE Healthcare, Piscataway, NJ, USA) equilibrated by TS buffer.

Protein concentrations were determined by the Bradford Protein Assay (Bio-Rad Laboratories, Hercules, CA, USA) using BioPhotometer Plus UV-vis equipment (Eppendorf, Hamburg, Germany). Bovine serum albumin was used as the standard.

### 4.5. SDS-Polyacrylamide Gel Electrophoresis and SYPRO Ruby Staining

The obtained protein fractions were subjected to standard SDS-PAGE, followed by SYPRO Ruby staining (Thermo Fisher Scientific, Waltham, MA, USA) and analyzed using the Luminescent Image Analyzer (ImageQuant LAS 4000, GE Healthcare) with All Blue (Bio-Rad Laboratories, Hercules, CA, USA) as the molecular weight marker.

### 4.6. Differential Scanning Fluorimetry

The differential scanning fluorimetry (DSF) experiment was performed using a real-time PCR system (Mx3005p; Agilent Technologies, Santa Clara, CA, USA) with MxPro software (Agilent Technologies) based on Niesen et al. [39]. Tubes containing 39 µL protein solution and 1 µL SYPRO Orange (Thermo Fisher Scientific) diluted tenfold with dimethyl sulfoxide (DMSO) were loaded into the PCR instrument and subjected to temperature scanning at 1 °C min^−1^ between 25 °C and 95 °C. The filter configurations were customized to accommodate the optimal excitation and emission wavelengths for SYPRO Orange (excitation, 492 nm; emission, 610 nm). The inflection points of the transition curves, indicating the protein melting temperatures (Tms), were estimated as described in an earlier report [53].

### 4.7. Fourier Transform Infrared Spectroscopy

Shrimp shell α-chitin (Sigma-Aldrich, St. Louis, MO, USA) was powdered in a Wiley mill (Thomas Scientific, Swedesboro, NJ, USA) to 250 μm particle size and used as chitin. Chitin was incubated with water, 0.1 M acetic acid (pH 2.8), 0.1 M Gly-HCl (pH 2.5), or 8 M urea. After incubation for 24 h at room temperature, chitin was filtrated, cleansed, and dried. The buffer-treated chitin and dried KBr were thoroughly ground to form pellets. FT-IR analysis was conducted using an FT-IR spectrophotometer (IRPrestige-21, Shimadzu Corporation, Kyoto, Japan).

### 4.8. Chitinase Enzymatic Assays

Chitinolytic activity was determined using a synthetic substrate, 4-NP-chitobioside (Sigma-Aldrich, St. Louis, MO, USA), essentially as described previously [12]. Chia unit definition was also reported previously [12].

### 4.9. Degradation of Chitin Beads, Colloidal and Crystalline Chitin Substrates by Chia

Chitin beads (10 µL) were incubated in a volume of 50 µL with or without purified Chia (4 mU) in 0.1 M acetic acid (pH 2.8) or 0.1 M Tris-HCl (pH 7.6) at 37 °C for 1 h. Colloidal and crystalline chitin were incubated in a volume of 50 µL containing purified Chia (4 mU) in 0.1 M Gly-HCl (pH 2.8) or 0.1 M acetic acid (pH 2.8), essentially as described previously [15,16,17]. Generated chitin fragments were analyzed by FACE as originally described by Jackson [41] and recently improved [42].

### 4.10. E. coli Expression Vectors and Preparation of Recombinant Fusion Proteins

Coding regions of the mature form of Chia cDNAs were amplified from chicken glandular stomach cDNA [16] by PCR using KOD Plus DNA polymerase (Toyobo Co., Ltd., Osaka, Japan) and oligonucleotide primers (Eurofins Genomics, Tokyo, Japan) anchored with the restriction sites for EcoRI and XhoI (Table S1), as described previously [12]. Amplified cDNA was digested with EcoRI and XhoI and cloned into the same sites of the pEZZ18/Protein A-mouse Chia-V5-His [12]. The entire nucleotide sequence of the resulting plasmid DNA [pEZZ18/PA-Chia] was confirmed by sequencing (Eurofins Genomics).

To express the CatD or CBD of chicken Chia as a recombinant fusion protein with Protein A and V5-His, these regions were amplified from the chicken full-length Chia-expressing plasmid DNA (pEZZ18/PA-Chia) using oligonucleotide primers (Table S1), as described previously [14]. Each amplified DNA was then digested with EcoRI and XhoI and subcloned into the pEZZ18 expression vector. The entire nucleotide sequence of the resulting plasmid DNAs [pEZZ18/PA-CatD or pEZZ18/PA-CBD] was confirmed by sequencing (Eurofins Genomics). The recombinant PA-Chia, PA-CatD, and PA-CBD (Figure S1) as well as PA were prepared as described previously [14].

### 4.11. Statistical Analysis

Biochemical data were compared by Student’s *t*-test. We carried out experiments in triplicate for the statistical analysis.

## 5. Conclusions

We showed that purifying native Chia from chitin columns using acetic acid could be utilized in various fields of agriculture and biomedicine as Chia-related supplementation.

## Supplementary Materials

Supplementary materials can be found at http://www.mdpi.com/1422-0067/19/2/362/s1.
