# Supplementary material for: Acidic Chitinase-Chitin Complex Is Dissociated in a Competitive Manner by Acetic Acid: Purification of Natural Enzyme for Supplementation Purposes
Source: Int J Mol Sci. 2018 Jan 25;19(2):362. doi: 10.3390/ijms19020362 (PMC5855584; doi:10.3390/ijms19020362)
Supplement: Supplementary file 1 [file ijms-19-00362-s001.pdf]

International Journal of *Molecular Sciences*

MDPI

Article

Acidic Chitinase-Chitin Complex Is Dissociated in a Competitive Manner by Acetic Acid: Purification of Natural Enzyme for Supplementation Purposes

Eri Tabata [1], Akinori Kashimura [1], Satoshi Wakita [1], Masayoshi Sakaguchi [1], Yasusato Sugahara [1], Yasutada Imamura [1], Hideaki Shimizu [2], Vaclav Matoska [3], Peter O. Bauer [3,4] and Fumitaka Oyama [1,*]

Protein A-chicken Chia-V5-His (PA-Chia)
618 amino acids, 67,820 dalton

```
AQHDEAVDNKFNKEQQNAFYEILHLPNLNEEQRNAFIQSLKDDPSQSANLLAEAKKLND
AQAPKVDNKFNKEQQNAFYEILHLPNLNEEQRNAFIQSLKDDPSQSANLLAEAKKLNDA
QAPKVDANSYVLSCYFTNWAQYRPGLGKYMPDNIDPCLCDHLIYAFAGMSNNEITTYEW
NDETLYKSFNGLKNQNGNLKTLLAIGGWNFGTAKFSTMVSTPENRQTFINSVIKFLRQY
QFDGLDIDWEYPGSKGSPSQDKGLFTVLVQEMLAAFEQEAKQVNKPRLMITAAVAAGLS
NIQAGYQIAELGKYLDYFHVMTYDFHGSWDGQTGENSPLYKGPADTGDLIYFNVDYAMN
YWKSNGAPAEKLLVGFPTYGHSYILKNPSDTAVGAPTSGPGPAGPYTRQSGFLAYYEIC
TFLDSGATQAWDAPQDVPYAYKSSEWVGYGNIKSFNIKIDWLKKNNYGGAMVWSLDMDD
FTGTFCKQGKYPLITTLKNALGQQSSSCVPPAQPNPPITAAPSTGSGSGSGSGSGSSGS
NTGSSGGSGFCAGKANGIYADPTNKSKFYNCNNGETFVQSCQAGLVFDSSCSCCNWAAR
GHPFEGKPIPNPLLGLDSTRTGHHHHHH
```

A. Protein A-chicken CatD-V5-His (PA-CatD)
543 amino acids, 60,587 dalton

```
AQHDEAVDNKFNKEQQNAFYEILHLPNLNEEQRNAFIQSLKDDPSQSANLLAEAKKLND
AQAPKVDNKFNKEQQNAFYEILHLPNLNEEQRNAFIQSLKDDPSQSANLLAEAKKLNDA
QAPKVDANSYVLSCYFTNWAQYRPGLGKYMPDNIDPCLCDHLIYAFAGMSNNEITTYEW
NDETLYKSFNGLKNQNGNLKTLLAIGGWNFGTAKFSTMVSTPENRQTFINSVIKFLRQY
QFDGLDIDWEYPGSKGSPSQDKGLFTVLVQEMLAAFEQEAKQVNKPRLMITAAVAAGLS
NIQAGYQIAELGKYLDYFHVMTYDFHGSWDGQTGENSPLYKGPADTGDLIYFNVDYAMN
YWKSNGAPAEKLLVGFPTYGHSYILKNPSDTAVGAPTSGPGPAGPYTRQSGFLAYYEIC
TFLDSGATQAWDAPQDVPYAYKSSEWVGYGNIKSFNIKIDWLKKNNYGGAMVWSLDMDD
FTGTFCKQGKYPLITTLKNALGQQSSSCVPPAQPNPPITAAARGHPFEGKPIPNPLLGL
DSTRTGHHHHHH
```

B. Protein A-chicken CBD-V5-His (PA-CBD)
232 amino acids, 24,970 dalton

```
AQHDEAVDNKFNKEQQNAFYEILHLPNLNEEQRNAFIQSLKDDPSQSANLLAEAKKLND
AQAPKVDNKFNKEQQNAFYEILHLPNLNEEQRNAFIQSLKDDPSQSANLLAEAKKLNDA
QAPKVDANSPSTGSGSGSGSGSGSSGSNTGSSGGSGFCAGKANGIYADPTNKSKFYNCN
NGETFVQSCQAGLVFDSSCSCCNWAARGHPFEGKPIPNPLLGLDSTRTGHHHHHH
```

Figure S1. Deduced amino acid sequences and their molecular masses of Protein A-chicken Chia-V5-His, Protein A-chicken CatD-V5-His and Protein A-chicken CBD-V5-His expressed in *E. coli*. The amino acid sequences are color coded. Yellow, mature form of truncated form of Protein A; Blue, Chia, CatD or CBD; Green, V5-His sequence.

International Journal of
Molecular Sciences

MDPI

Table S1. Forward and reverse primers used to construct the *E. coli*-expression vectors.

1. Construction of pEZZ18/ Protein A-chicken Chia-V5-His

EcoRI chicken Chia_Fw

CATG<u>GAATTC</u>GTATGTGCTGTCATGCTATTTCACCA

XhoI chicken Chia_Rv

GTGAC<u>CTCGAG</u>GTGCCCAGTTGCAGCAGGAACAGCTG

2. Construction of pEZZ18/ Protein A-chicken CatD-V5-His

EcoRI_chicken Chia_Fw

CATG<u>GAATTC</u>GTATGTGCTGTCATGCTATTTCACCA

XhoI_chicken Chia CatD_Rv

ccttcgaatgggtgac<u>CTCGAG</u>gAGCTGCAGTGATGGGAGGATTGGGC

3. Construction of pEZZ18/ Protein A-chicken CBD-V5-His

EcoRI chicken Chia CBD Fw

cgccgaaagtagacgc<u>GAATTC</u>gCCTAGCACTGGAAGTGGGAGTGGGA

XhoI_Chia_Rv

GTGAC<u>CTCGAG</u>GTGCCCAGTTGCAGCAGGAACAGCTG
